# Crystal Structure Evolution of UHMWPE/HDPE Blend Fibers Prepared by Melt Spinning

**DOI:** 10.3390/polym9030096

**Published:** 2017-03-09

**Authors:** Fei Wang, Lichao Liu, Ping Xue, Mingyin Jia

**Affiliations:** Institute of Plastic Machinery and Engineering, Beijing University of Chemical Technology, Beijing 100029, China; wangfei_987321@163.com (F.W.); liulc@mail.buct.edu.cn (L.L.); jiamy@mail.buct.edu.cn (M.J.)

**Keywords:** UHMWPE, HDPE, fibers, blend, melt spinning

## Abstract

Ultra-high molecular weight polyethylene (UHMWPE) and high-density polyethylene (HDPE) blend fibers with the highest tensile strength of 1.13 GPa were prepared by a melt spinning process. The mechanical behavior and crystal structure of the as-spun filaments and fibers were studied by differential scanning calorimetry (DSC), scanning electron microscopy (SEM), X-ray diffraction (XRD), sound velocity orientation testing, and tensile testing. The orientation degree, crystallinity, tensile strength, and initial modulus of the fibers increased with the increasing of the draw ratios. The grain size was shortened in the radial direction and elongated in the axial direction. The results suggested that the improvement of the tensile strength and initial modulus was a result of the compact crystal structure formed by slender grains composed of highly oriented molecular chains. Blending with HDPE could improve the formation of a slender and compact crystal structure, and the tensile strength and initial modulus of the blend fibers were higher.

## 1. Introduction

Ultra-high molecular weight polyethylene (UHMWPE) fibers were first developed by Smith et al. using a gel spinning method in 1980, with a Young’s modulus >90 GPa, a tensile strength >3 GPa, and a strain at break of 6% [1]. These fibers have been widely used in medical, aerospace, sports, transportation, military, engineering, and other fields, replacing other fibers, such as Kevlar and carbon fiber [2]. At present, the tensile strength of UHMWPE fibers prepared by the gel spinning method can reach more than 4 GPa [3]. However, the organic solvent, extracting agent, and relevant devices have many issues, such as high cost, environmental pollution, and low production efficiency [4]. The melt spinning method makes up for the disadvantages that exist in the gel spinning method and is widely used in the industrial production of polyester (PET) fibers [5,6,7,8,9], polypropylene (PP) fibers [10,11,12,13], high-density polyethylene (HDPE) fibers [14], and other fibers. However, the extremely long molecular chains of UHMWPE are entangled together, resulting in a high viscosity after melting. Melt fracture will occur on the surface of the UHMWPE as-spun filaments in the spinning process, and defects of the as-spun filaments caused by melt fracture will reduce the mechanical properties of the fibers, so there are challenges to prepare UHMWPE fibers by the melt spinning method. Therefore, UHMWPE raw materials need to be modified to meet the processing requirements of melt spinning [15]. Adding nanomaterials and blending with low-molecular-weight polymers are the two main methods to modify the flowability of UHMWPE used in the melt spinning method. Adding montmorillonite (MMT) could improve the extension of the molecular chains of the UHMWPE, so the UHMWPE/MMT nanocomposite fibers were prepared successfully using the melt spinning method [15,16,17]. TiO_2_ could embed in the wall of the capillary, thereby avoiding wall slip, so that the spinning ran smoothly [18]. Blending with low-density polyethylene (LDPE), linear low-density polyethylene (LLDPE) [19], and polyolefin [20] could improve the flowability of UHMWPE. In this study, the blending material used was HDPE with a melt flow index (MFI) of 0.9 g/10 min. Blend fibers were prepared by melt spinning and the highest tensile strength of the blend fibers was 1.13 GPa.

Researchers have studied the structural evolution of UHMWPE fibers prepared by gel spinning, and it has been proven that the excellent mechanical properties of gel-spun UHMWPE fibers are due to the internal regular shish-kebab structure [21,22,23]. The microstructures of the low-molecular-weight polymer/nanomaterial fibers have been studied in many papers. It was found that the molecular chain orientations and the crystallization ability of the fibers were influenced by adding inorganic nanomaterials [10,11,12,13,14]. The microstructures of the low-molecular-weight polymer blend fibers have also been studied by some papers. Takahashi et al. reported that the orientation of PP/PA6 blend fibers was improved [24], and Xiao et al. reported that the crystallization ability of the PP/PVA blend fibers was improved [25]. Rwei reported that blending PEN into PET would suppress the stress-induced orientation and, furthermore, decrease the stress-induced crystallization of PET filaments [9]. Kim et al. reported that the TLCP/PEN/PET blend fibers had more perfect crystalline structures [26]. However, there are few studies on the structural evolution of UHMWPE fibers prepared by melt spinning. In the study of Zhang et al. [15,16], the structural evolution of the UHMWPE/MMT fibers was reported. They found that the molecular chains of UHMWPE could be easily disentangled by adding MMT. In the study reported here, the modified material used was conventional polyethylene, which was HDPE. It has been proven that the molecular chains of UHMWPE and HDPE can affect each other, improving the structure of the blends. Zuo et al. reported that middle-molecular-weight polyethylene (MMWPE), as a fluidity modifier, could help UHMWPE molecular chains to slip and orient [27]. Shen and Zuo reported that the UHMWPE molecular chains became more easily disentangled when blending with HDPE in the melt processing [28,29]. The crystallization behavior of the blends is also improved. The mobility and crystal structure of the UHMWPE used for melt processing are improved significantly with the addition of HDPE. Zuo et al. reported that co-crystallization occurred in the blends of UHMWPE and HDPE, and adding MMWPE could increase the compatibility of the two phases and the co-crystallization between HDPE and UHMWPE [27]. Salkhi reported that in blends of UHMWPE and HDPE, the crystallization of UHMWPE could induce the crystallization of HDPE at higher temperatures, so that the crystallization rates of the blends were lower than those of the neat materials [30]. Jaggi et al. reported that the crystallite size increased with the increase of UHMWPE content in the HDPE/UHMWPE blend matrix [31]. However, few attempts have been made to study the melt spinning of UHMWPE/HDPE blend fibers [18] and the crystal structural evolution of the UHMWPE/HDPE blend fibers has not been reported by any study.

Therefore, the aim of this work is to prepare UHMWPE/HDPE blend fibers with excellent mechanical properties by melt spinning and to study the mechanical properties and microstructure of blend fibers by means of differential scanning calorimetry (DSC), scanning electron microscopy (SEM), sound velocity orientation test, wide-angle X-ray diffraction (WAXD) and tensile tests. The crystal structural evolution and its effect on the mechanical properties of the UHMWPE/HDPE blend fibers prepared by melt spinning method are discussed.

## 2. Materials and Methods

### 2.1. Materials

UHMWPE resin (GUR4012, Celanese Corporation, Nanjing, China) used in this study is associated with a weight-average molecular weight (*M*_w_) of 1.5 × 10^6^. HDPE (5000S, Yanshan Petrochemical Company, Beijing, China) used in this study has MFI of 0.9 g/10 min and *M*_w_ of 1.64 × 10^5^.

### 2.2. Preparation of UHMWPE/HDPE Blend Fibers

HDPE particles and UHMWPE powder were mixed in a high-speed mixer at room temperature with a rotating speed of 1440 rpm, and a mixing time of 10 min. In our trial experimental study, the mass ratios of UHMWPE and HDPE we used were 9:1, 8:2, 7:3, 6:4, and 5:5. The preliminary results showed that the blend fibers with the mass ratio of 6:4 had the best processability and tensile strength. Therefore, in this study we chose the blend materials with the mass ratio of 6:4 and pure UHMWPE materials to study the effect of HDPE on the crystal structure evolution of the as-spun filaments and fibers. The relevant parameters are listed in Table 1. Blends of UHMWPE and HDPE are referred to as U/H, and the neat UHMWPE is referred to U. The mixed materials were granulated using a co-rotating twin-screw extruder with a diameter of 20 mm and an aspect ratio (L/D) of 45. The screw speed was 200 r/min, and the temperature of the five zones was 160–300 °C.

The melt was extruded using a self-made special single-screw extruder, and the as-spun filaments were collected by the winder. The initial draw ratios from 1 to 9 were obtained by changing the take-up speed of the winder. The temperature of the extruder was set at 160 to 300 °C. The diameter of the spinneret hole was 0.6 mm, and the temperature of the spinneret was 310 °C. The U sample was the reference group. The obtained as-spun filaments with the initial draw ratio of 9 were later hot drawn at draw ratios of 3, 6, 9, 12, and 15 under 80 °C to obtain fibers having a certain level of specific mechanical properties.

### 2.3. Analytical Methods

The thermal properties of the as-spun filaments and fibers were analyzed by a differential scanning calorimetry (DSC2, Mettler Toledo, Greifensee, Switzerland). Typical sample weights used were ~5 mg. A heating rate of 10 °C/min and a temperature range from 25 to 250 °C was selected. The specimens were always tested in a nitrogen environment. The crystallinity of the samples can be obtained through Equation (1):

(1) $$X_{c}=\frac{\Delta H_{m}}{\Delta H_{m}^{0}}\times 100\%$$

where $X_{c}$ is the degree of crystallinity evaluated by the DSC method, $\Delta H_{m}$ is the melting enthalpy of the sample and $\Delta H_{m}^{0}$ is the melting enthalpy of a 100% crystalline sample and is taken as 290 J/g as published in the literature [32,33].

Wide-angle X-ray diffraction experiments of (110), (200), and (020) planes were carried out using an X-ray diffractometer (Bruker AXS D8 Advance diffractometer, Bruker, Billerica, MA, USA, Cu Kα radiation, λKαl = 1.542 Å, scanning from 5° to 50° in 2θ at a scanning speed of 10°/min); wide-angle X-ray diffraction experiments of the (002) plane were carried out using an X-ray diffractometer (XRD, EMPYREAN, PANalytical Co., Almelo, The Netherlands, Cu Kα radiation, scanning from 70° to 78° in 2θ at a scanning speed of 0.013°/step). 2θ was fixed on the scanning curve maxima (21.4°). Azimuth angle scanning was carried out in the range of –90° to 90° with 0.1°/min. The two-theta angle was calibrated according to the diffraction position of standard Si powder. The grain size (*L*_hkl_) in the direction perpendicular to the set of lattice planes was calculated by the Scherrer equation:

(2) $$L_{\mathrm{hkl}}=\frac{K\lambda }{H\cos\theta }$$

where λ is the wavelength of X-ray; θ is the Bragg angle; *H* is the half high width of diffraction peak (unit in radian). The degree of orientation in the crystallinity phases of fibers can be obtained through Equations (3) and (4):

(3) $$f_{c}=\frac{1}{2}\left(3\cos^{2}\varphi -1\right)$$

(4) $$\cos^{2}\varphi =\frac{\int_{-\frac{\pi }{2}}^{\frac{\pi }{2}}I_{\mathrm{hkl}}{\sin\varphi \cos}^{2}\varphi d\varphi }{\int_{-\frac{\pi }{2}}^{\frac{\pi }{2}}I_{\mathrm{hkl}}\sin\varphi d\varphi }$$

where *f*_c_ is orientation degree of the crystalline phase, and it takes the value 0, 1, and −1/2 with no orientation, perfect orientation, and perfect normal orientation in the drawing direction, respectively. *I*_hkl_ versus φ data can be obtained by the azimuthal scanning. *I*_hkl_ is the intensity of a specific plane, and φ is the azimuthal angle.

The sound velocity orientation test was performed using a SCY-III type of sound velocity orientation instrument (Donghua University, Shanghai, China), and the sound velocity value was calculated by using the double length method. The sound velocity orientation factor of the samples can be obtained through Equation (5):

(5) $$f_{s}=\left(1-\frac{C^{2}}{C_{m}^{2}}\right)\times 100$$

where C is the sound velocity of the sample, $C_{m}$ is the sound velocity of the fiber with random orientation of polyethylene and is taken as 1.65 km/s [34].

The mechanical properties of the fibers were evaluated using a YG001A-1 fiber electronic strength tester (Hongda Fangyuan Electric Co., Ltd., Taicang, China). The length of the fiber samples was set as 20 mm, and the extension rate was 10 mm/min. The fiber diameter was measured using a SMART biological microscope. Tests have been performed on at least five specimens for each sample and the average values have been reported along with the standard deviation.

The morphology of fibers was studied by using a S4700 scanning electron microscopy (SEM, Hitachi Ltd., Tokyo, Japan) with an acceleration voltage of 20 kV.

## 3. Results and Discussion

### 3.1. Sound Velocity Orientation Studies

**Figure 1:**
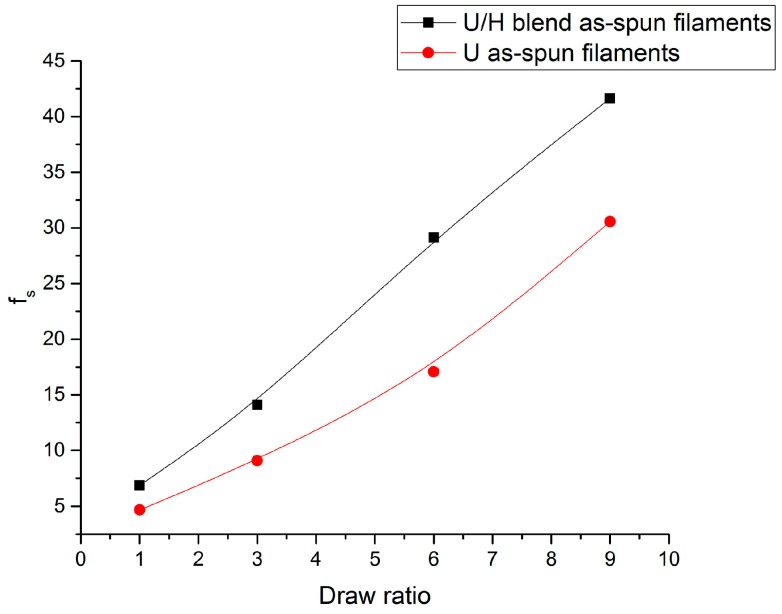
The orientation degree of the as-spun filament samples.

Figure 1 presents the orientation degree of the molecular chains (*f*_s_) of the as-spun filament samples at different initial draw ratios. As can be seen in Figure 1, with the initial draw ratios increasing from 1 to 9, the *f*_s_ values of the U/H blend as-spun filament samples and U as-spun filament samples increased from 6.89 to 41.65 and from 4.68 to 30.56, respectively. In the spinning line, the motion unit of the as-spun filaments is the whole molecular chain, and molecular chains are stretched mainly in the melting state. The orientation degree of chain segments is lower, so the sound velocity and sound velocity factor of the as-spun filaments cannot reach much higher. MMWPE, as a fluidity modifier, can help UHMWPE molecular chains to slip and orient [27], and blending with HDPE can make the disentanglement of the UHMWPE molecular chains easier in the melt processing [28,29], so that the orientation degree of the U/H blend as-spun filaments is higher than that of the U as-spun filaments with the same initial draw ratios.

**Figure 2:**
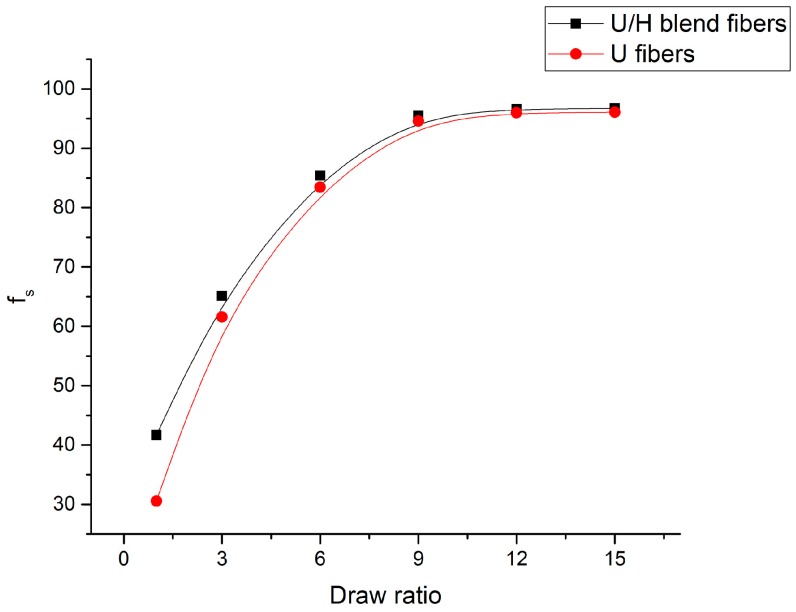
The orientation degree of the fiber samples.

The *f*_s_ values of the fiber samples at different hot-draw ratios are presented in Figure 2. The *f*_s_ values of the U/H blend fiber samples and the U fiber samples increased rapidly from 41.65 to 85.41 and from 30.56 to 83.44, respectively, with the hot-draw ratios increasing from 1 to 6. Fibers in the hot drawing process are in a highly elastic state, and the motion units are chain segments. They are easily stretched in the beginning of the hot drawing process, so the orientation degree of the fibers increased significantly when the hot-draw ratios increased from 1 to 6. The *f*_s_ values of the U/H blend fiber samples and the U fiber samples increased slightly from 85.41 to 96.73 and from 83.44 to 96.07, respectively, with the hot-draw ratios increasing from 6 to 15. The increasing rate of the *f*_s_ values of both the U/H fiber samples and the U fiber samples dropped significantly when the hot-draw ratios increased from 6 to 15, indicating that molecular chain segments are already highly oriented with the hot-draw ratios reaching 6. Therefore, the molecular chain segments were stretched slightly as the hot-draw ratios increased to greater than 6. The orientation degree of the molecular chains of the U/H blend fibers was higher than that of the U fibers at the same hot-draw ratios, suggesting that the relatively shorter molecular chains of HDPE can also improve the orientation of the chain segments of the extremely long molecular chains of UHMWPE in the hot drawing process.

### 3.2. SEM Studies

**Figure 3:**
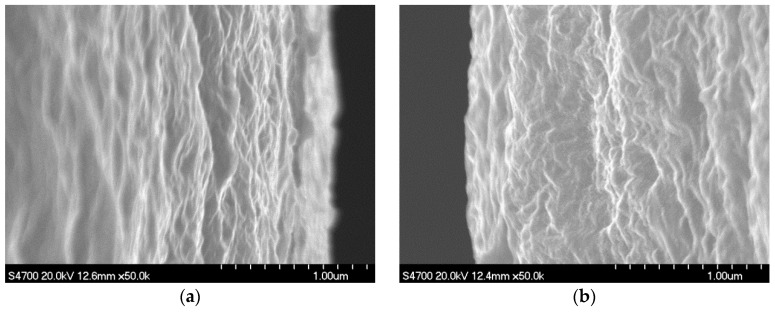
SEM images of as-spun filaments: (**a**) U/H blend as-spun filament samples with the initial draw ratio of 9; and (**b**) U as-spun filament samples with the initial draw ratio of 9.

**Figure 4:**
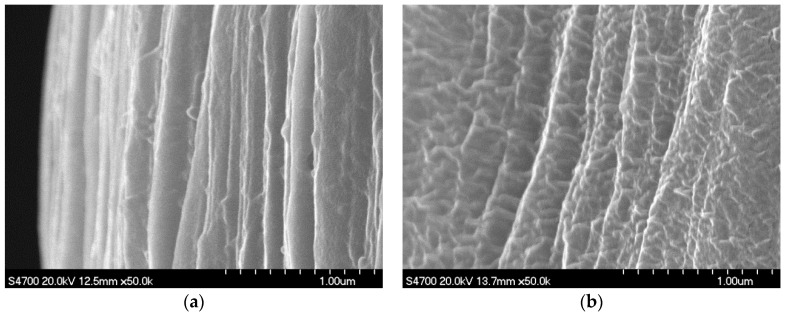
SEM images of fiber samples: (**a**) U/H blend fiber samples with the hot-draw ratio of 15; and (**b**) U fiber samples with the hot-draw ratio of 15.

SEM images of as-spun filament samples and fiber samples are presented in Figure 3 and Figure 4, respectively. The surface of the U/H blend as-spun filament samples and fiber samples was smoother and had a higher orientation than that of the U as-spun filament samples and fiber samples, which indicates that blending with HDPE can improve the surface quality of the as-spun filaments and fibers. That is, HDPE improves the processing performance of UHMWPE for the melt spinning and hot drawing process.

### 3.3. DSC Analyses

**Figure 5:**
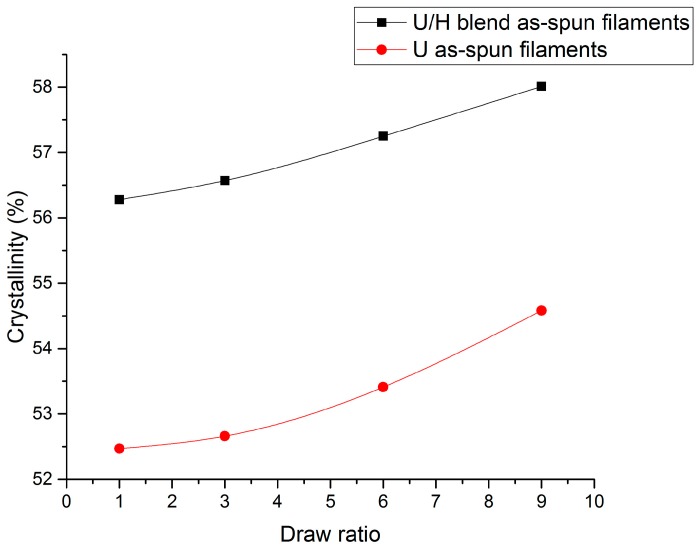
The crystallinity values of as-spun filament samples with different initial draw ratios.

The DSC analysis data of as-spun filament samples are presented in Figure 5. The crystallinity of the U/H blend as-spun filament samples and the U as-spun filament samples increased from 56.28% to 58.01% and from 52.47% to 54.58%, respectively, as the initial draw ratios increased from 1 to 9. The whole molecular chains slip relative to each other in the spinning line, and they are arranged slightly. Therefore, the crystallinity of the as-spun filaments increased in the spinning process. The crystallinity of the U/H blend as-spun filaments was higher than that of the U as-spun filaments at the same initial draw ratios. The higher crystallinity of the blend as-spun filament samples is a result of the better-oriented molecular chains.

**Figure 6:**
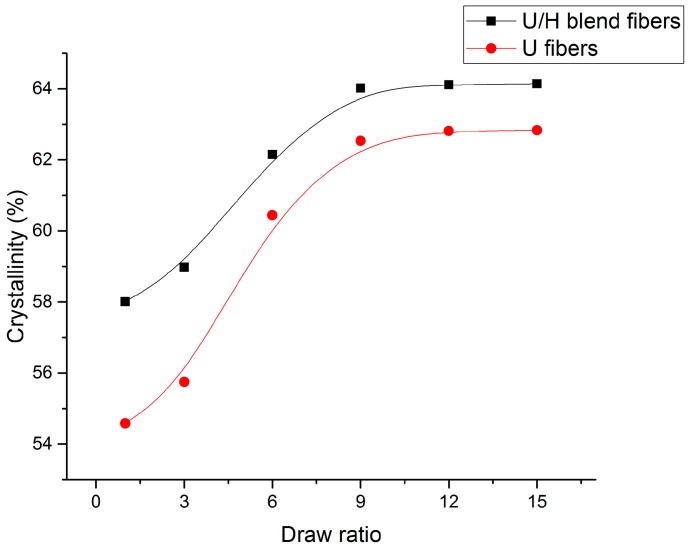
The crystallinity values of fiber samples with different hot-draw ratios.

The crystallinities of the U/H blend fiber samples and the U fiber samples are presented in Figure 6. As for Figure 6, the crystallinity of the U/H fiber samples and the U fiber samples increased rapidly from 58.01% to 64.02% and from 54.58% to 62.53%, respectively, as the hot-draw ratios increased from 1 to 9. The crystallinity of the U/H blend fiber samples and the U fiber samples increased slightly from 64.02% to 64.14% and from 62.53% to 62.83%, respectively, with the hot-draw ratios increasing from 9 to 15. The rapid increase of the crystallinity at lower hot-draw ratios is possibly a result of the new crystalline region formed by partial molecular chains with straightened chain segments in the amorphous area. The crystallinity of the U/H blend fiber samples was higher than that of the U fiber samples, indicating that adding relatively short molecular chains of HDPE can form straighter chain segments in the amorphous area, so the crystallinity increased.

**Figure 7:**
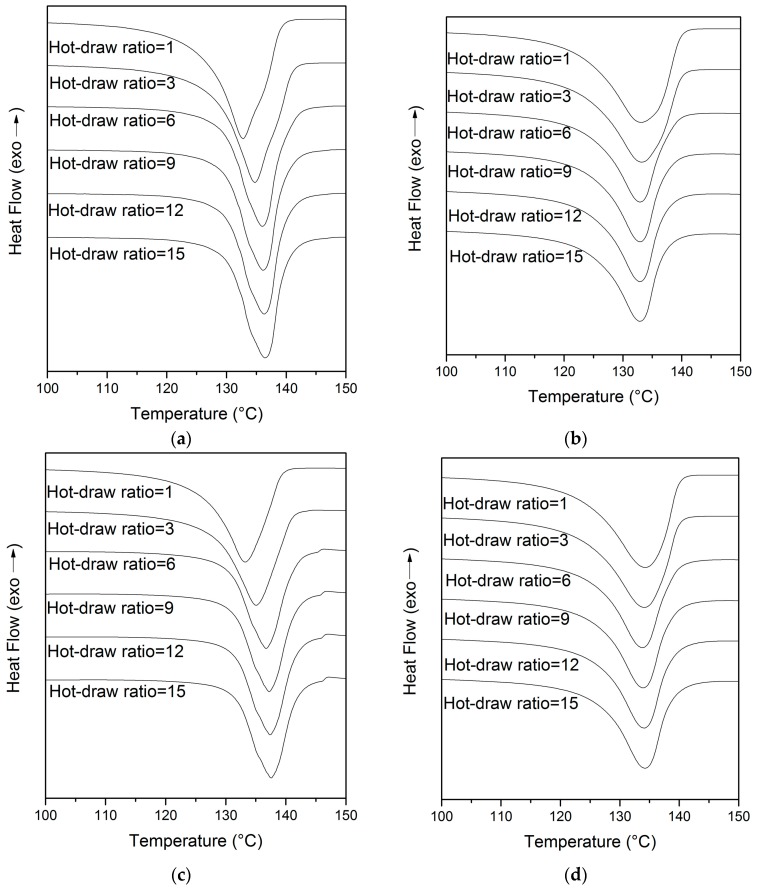
DSC thermograms of fibers with different draw ratios: (**a**) the first heating curves of the U/H blend fiber samples; (**b**) the second heating curves of the U/H blend fiber samples; (**c**) the first heating curves of the U fiber samples; and (**d**) the second heating curves of the U fiber samples.

The DSC thermograms of the U/H and U fiber samples are presented in Figure 7. As shown in Figure 7a,c, the curves were shifted to the right as the hot-draw ratios increased from 1 to 15. The melting peak temperature of the U/H blend fiber samples and the U fiber samples increased from 131.12 to 135.18 °C and from 132.43 to 136.42 °C, respectively, with the hot-draw ratios increasing from 1 to 6, and increased slightly when the hot-draw ratio was higher than 6. The reason for the increase of the melting peak temperature is due to the new crystallization zone, which requires a higher temperature for melting, formed by the extended molecular chains of the fibers. The curves of both the U/H blend fiber samples and the U fiber samples were smooth when the draw ratio was lower than 6, and little shoulders appeared at the left side of the melting peak with the hot-draw ratios increasing from 9 to 15. The appearance of the shoulders is due to the crystal structure formed by the extended molecular chains of the fibers. 

The second heating curves of the U/H and U blend fibers are presented in Figure 7b,d. As can be seen in Figure 7b,d, at the same draw ratios, the second heating curves of the fibers became smoother than the first heating curves, and the curves changed very little as the hot-draw ratios increased from 1 to 15. Moreover, the shoulders disappeared in the second heating curves. The smoother and similar curves with different drawing ratios, indicating that the crystal structure formed by extended molecular chains in the spinning and hot drawing process, disappeared as the thermo-history was eliminated. In the second heating, the peak melting temperature of the two fiber samples was almost unchanged with the increase of the hot-draw ratios, and the melt peak temperature of the U/H blend fibers was ~133 °C and that of the U fibers was ~134 °C. The crystallinity of the U/H blend fibers and the U fibers with different hot-draw ratios was less than 53%, and was almost unchanged with the increase of the hot-draw ratios. The melting peak temperatures and the crystallinity of the second heating process reflect the properties of the microstructures formed during the cooling process, in which the crystal structures formed in the spinning process and hot drawing process are broken. The almost unchanged melting peak temperature and crystallinity of both the U/H blend fibers and U fibers indicate that HDPE can improve the orientation of the molecular chains and the formation of the crystal structure in a uniaxial stretching process, and cannot improve them in a normal cooling process.

### 3.4. WAXD Analyses

**Figure 8:**
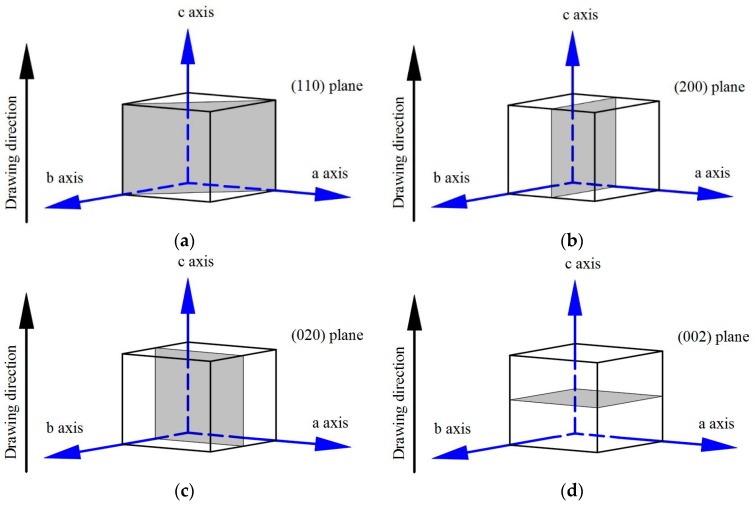
Plane orientation inside of orthombic unit cell: (**a**) (110) plane; (**b**) (200) plane; (**c**) (020) plane; and (**d**) (002) plane.

There are four diffraction peaks from the orthorhombic crystal structure of polyethylene that were of special interest: the (110), (200), (020), and (002). The (110) and (200) peaks appeared at 2θ angles from 13° to 27°, the (020) peak appeared at 2θ angles from 33° to 38°, and the (002) peak appeared at 2θ angles from 72° to 76° [35]. A schematic diagram of the (110), (200), (020), and (002) planes is shown in Figure 8. Grain size (*L*_hkl_) in the normal direction of the lattice planes was calculated by the Scherrer equation.

**Figure 9:**
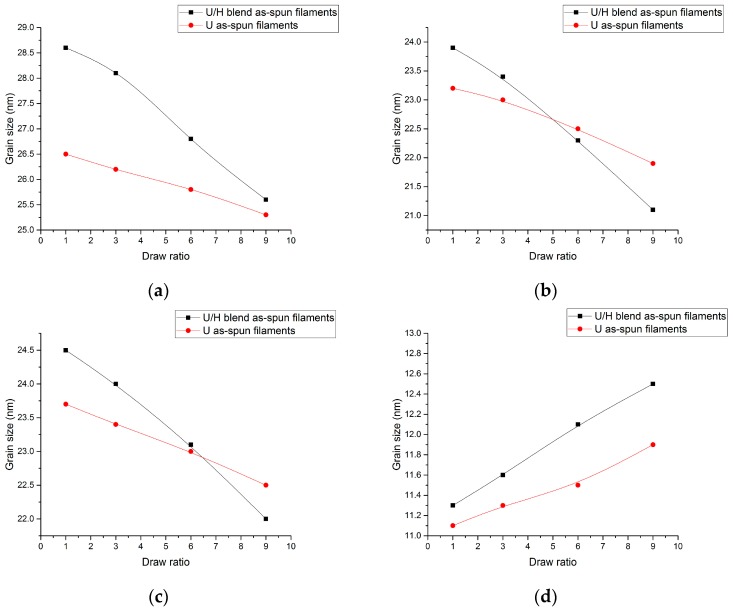
The grain size of the as-spun filament samples: (**a**) (110) plane; (**b**) (200) plane; (**c**) (020) plane; and (**d**) (002) plane.

The grain sizes in the normal directions of the (110), (200), (020), and (002) planes of the filament samples are presented in Figure 9. As can be seen in Figure 9, *L*_110_, *L*_200_, and *L*_020_ decreased and *L*_002_ increased as the initial draw ratios increased. It is indicated that the crystal was elongated along the drawing direction and shortened in the direction perpendicular to the drawing direction. *L*_110_, *L*_200_, and *L*_020_ of the U/H blend as-spun filaments were higher than those of the U as-spun filament samples with the initial draw ratio of 1. As the initial draw ratio reached 9, *L*_200_ and *L*_020_ of the U/H blend as-spun filament samples were lower than those of the U as-spun filament samples, and only *L*_110_ of the U/H blend as-spun filament samples was still higher than that of the U as-spun filament samples. *L*_002_ of the U/H blend as-spun filament samples was higher than that of the U as-spun filament samples with the same initial draw ratio, but the difference between them was larger as the initial draw ratio reached 9. It is suggested that blending with HDPE can effectively reduce the crystal size in the direction perpendicular to the initial drawing direction and elongate the crystal in the initial drawing direction. 

**Figure 10:**
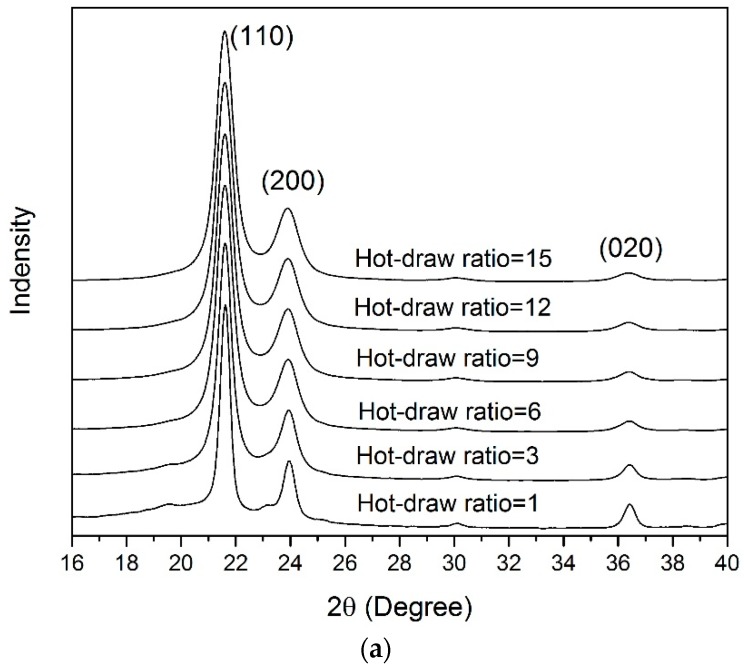

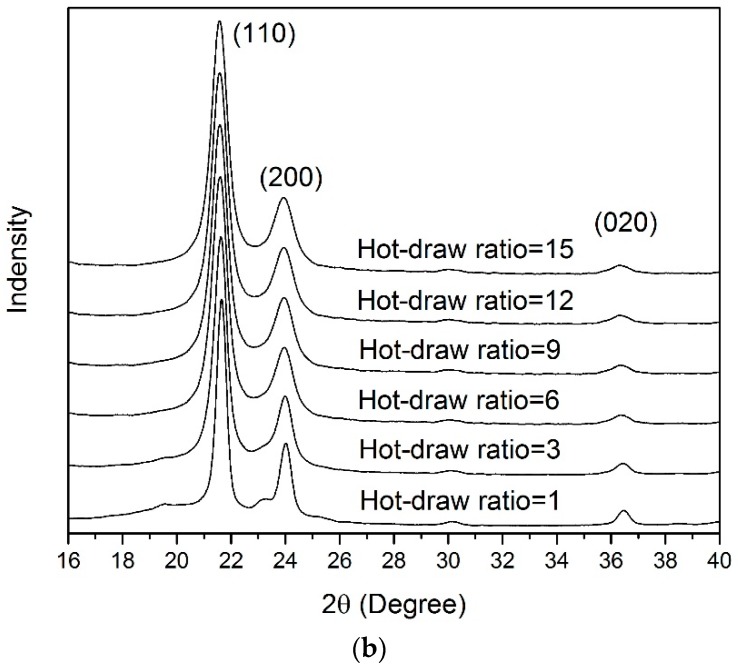
WAXD patterns of fiber samples with different hot-draw ratios: (**a**) U/H blend fiber samples; and (**b**) U fiber samples.

The X-ray diffraction patterns of the U/H blend fiber samples and the U fiber samples are presented in Figure 10. Three typical characterized peaks of (110), (200), and (020) crystal planes with 2θ angles of about 21°, 24°, and 36° were obtained in Figure 10. The full width at half maximum (FWHM) increased gradually with the increase of the hot-draw ratios.

**Figure 11:**
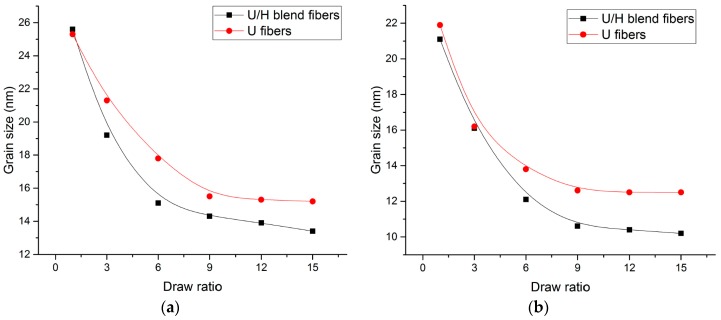

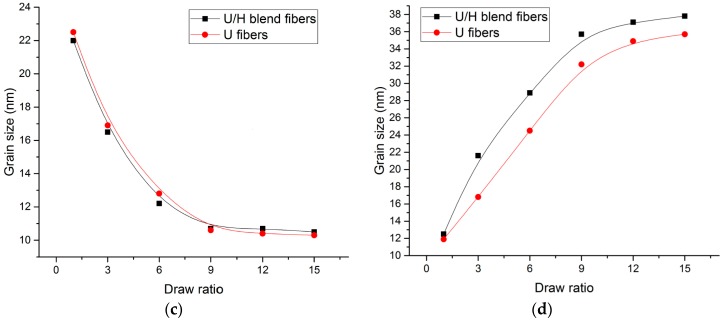
The grain size of the fiber samples: (**a**) (110) plane; (**b**) (200) plane; (**c**) (020) plane; and (**d**) (002) plane.

**Figure 12:**
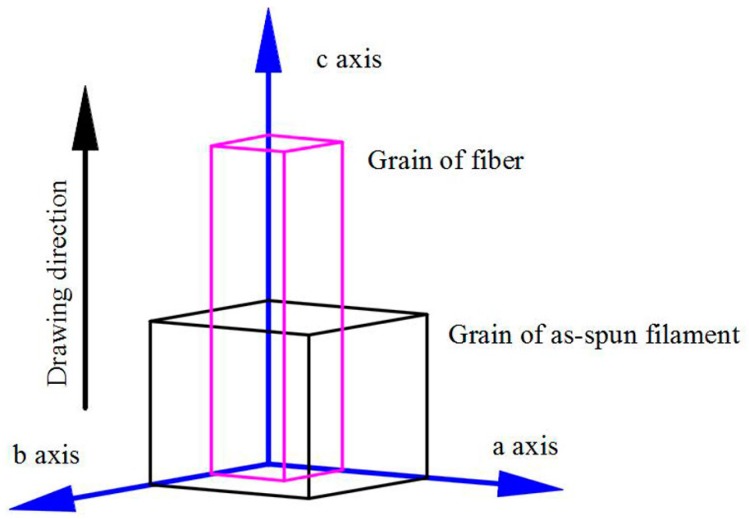
Deformation of the crystal structure during hot drawing process.

Figure 11 presents the grain sizes in the normal direction of the (110), (200), (020), and (002) planes, respectively. *L*_110_, *L*_200_, and *L*_020_ of the two fiber samples decreased rapidly as the hot-draw ratios increased from 1 to 6, and decreased slightly as the hot-draw ratios increased from 9 to 15. The L_002_ values of the two fiber samples increased rapidly as the hot-draw ratios increased from 1 to 9 and increased slightly as the hot-draw ratios increased from 12 to 15. In the hot drawing process, the crystal structures are stretched in the drawing direction (the normal direction of the (002) plane) and compacted in the radial direction (the normal direction of the (200) and (020) planes) as the chain segments orient along the drawing direction, so the grains gradually become slender as the hot drawing process proceeds (Figure 12). At the same time, the slender crystal structures are more easily arranged in the drawing direction under the stretching process. Therefore, the gaps between the grains are smaller. With the same hot-draw ratios, *L*_110_ and *L*_200_ of the U/H blend fiber samples were lower than those of the U blend fiber samples, L_002_ of the U/H blend fiber samples was higher than that of the U fiber samples, and *L*_020_ of the U/H blend fiber samples was basically the same as the U fiber samples. The formation of the more slender grain shape of the U/H blend fibers is probably because the relatively short molecular chains of HDPE improve the orientation of the chain segments. The crystal grains formed by the higher-oriented molecular chains have a larger size in the axial direction and a smaller size in the radial direction.

**Figure 13:**
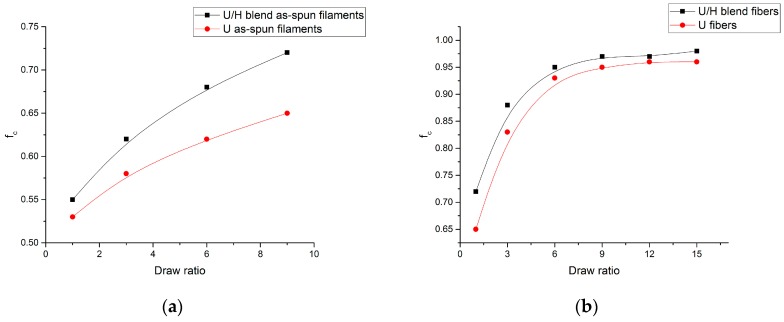
Crystal orientation of as-spun filament and fiber samples: (**a**) as-spun filament samples; and (**b**) fiber samples.

Figure 13 shows the crystalline orientation (*f*_c_) values of as-spun filament samples and fiber samples with different draw ratios. As shown in Figure 13a, the *f*_c_ values of the U/H blend as-spun filament samples increased from 0.55 to 0.72, and the *f*_c_ values of the U as-spun filament samples increased from 0.53 to 0.65 with the increase of the initial draw ratios. The initial drawing process mainly occurs in the melt state of the as-spun filaments, and the molecular chain slippage cannot improve the crystalline orientation to a higher level. As can be seen in Figure 13b, the *f*_c_ values of the U/H blend fiber samples and the U fiber samples increased rapidly from 0.72 to 0.95 and from 0.65 to 0.93, respectively, as the hot-draw ratios increased from 1 to 6. Then the *f*_c_ values of the U/H blend fiber samples and the U fiber samples increased slightly from 0.95 to 0.98 and from 0.93 to 0.95, respectively, as the hot-draw ratios increased from 6 to 15. The variation trend of *f*_c_ is similar to that of *f*_s_, indicating that the crystalline orientation is affected by the molecular chain orientation in the drawing process. Since the main motion in the initial drawing process is due to molecular chain slippage, the crystal structures cannot be significantly aligned in the drawing direction. In the hot drawing process, crystal structures are stretched in the high-elastic state, in which chain segments are stretched in the hot drawing direction and the grain size becomes smaller in the radial direction. Therefore, the *f*_c_ values of fibers can reach a high level in the hot drawing process. The relatively short molecular chains of HDPE improve the orientation of the chain segments in the blend fibers, and then the crystal structures are more likely to align in the drawing direction. Therefore, the *f*_c_ values of the U/H fiber samples were higher than those of the U fiber samples at the same hot-draw ratios.

### 3.5. Tensile Test

**Figure 14:**
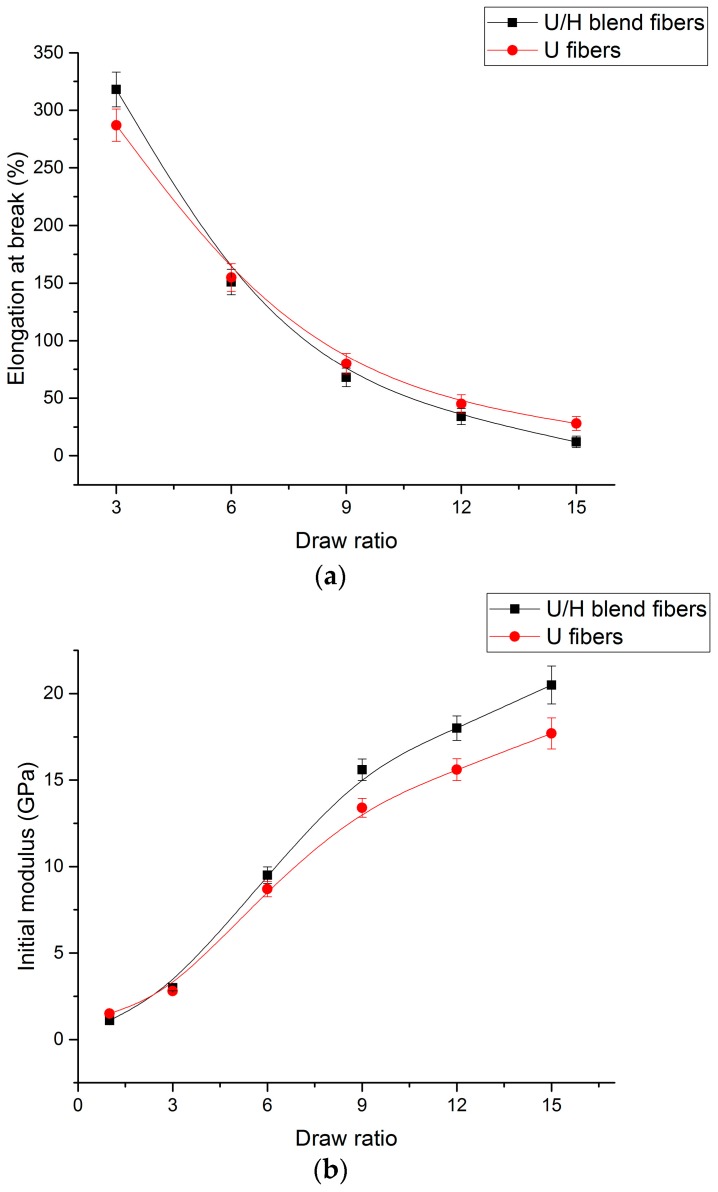

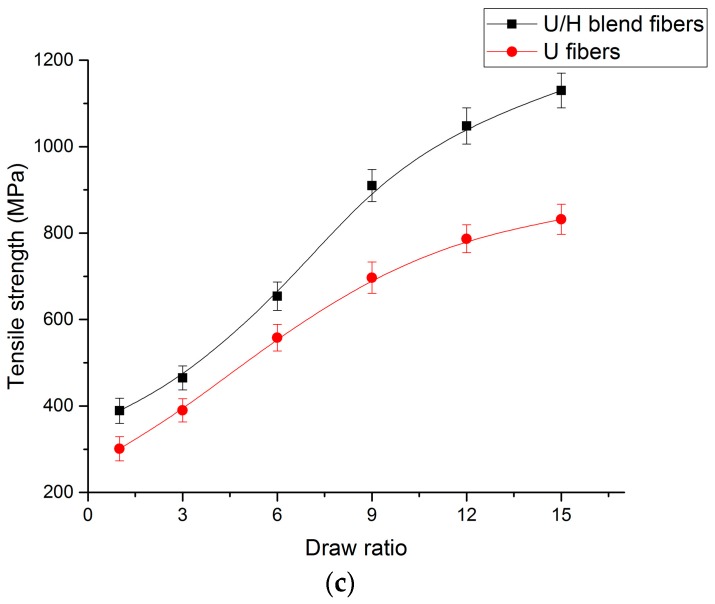
Mechanical properties of fibers: (**a**) elongation at break; (**b**) initial modulus; and (**c**) tensile strength.

The take-up speeds of the winder and the as-spun filament diameters with different initial drawing ratios are presented in Table 2, and the fiber diameters with different hot-draw ratios are presented in Table 3. The mechanical properties obtained from the tensile test are presented in Figure 14. As can be seen in Figure 14a, the elongation at break of both the U/H blend fibers and the U fibers decreased rapidly from 318% to 68% and 287% to 80%, respectively, as the hot-draw ratios increased from 3 to 9, and then decreased slowly from 68% to 12% and from 80% to 28%, respectively. The elongation at break of the U/H blend fibers was higher than that of the U fibers with the hot-draw ratio of 3, and began to be lower than that of the U fibers when the hot-draw ratio was higher than 6. Figure 14b presents the initial modulus of the U/H blend fiber samples and U fiber samples. The initial modulus of the U/H blend fibers and U fibers increased from 1.1 to 15.6 GPa and 1.5 to 13.4 GPa, respectively, as the hot-draw ratios increased from 1 to 9, and increased from 15.6 to 20.5 GPa and from 13.4 to 17.7 GPa, respectively, as the hot-draw ratios increased from 9 to 15. Similar to the elongation at break, the initial modulus of the U/H blend fibers was lower than that of the U fibers with a lower hot-draw ratio (<3), and began to be higher than that of the U fibers when the hot-draw ratio was higher than 6. Figure 14c presents the tensile strength of the U/H blend fibers and the U fibers with different hot-draw ratios. The tensile strength of the U/H and the U fibers increased rapidly from 389 to 910 MPa and from 301 to 697 MPa, respectively, with the hot-draw ratios increasing from 1 to 9, and then increased slowly from 910 to 1130 MPa and from 697 to 832 MPa, respectively, as the hot-draw ratios increased from 9 to 15. The tensile strengths of the U/H blend fibers were slightly higher than those of the U fibers with a lower hot-draw ratio (<3), and with the increase of the hot-draw ratio, the difference between the tensile strengths of the two kinds of fiber increased.

Although the molecular chain orientation degree, crystal structure orientation degree, and crystallinity of the as-spun filaments are improved by the spinning process, they are still at a low level, and the crystal grains are not sufficiently elongated in the axial direction. At a lower hot-draw ratio, the effect of the HDPE on the blend fibers in the hot drawing process was not reflected, so the elongation at break of the U/H blend fibers was slightly higher than that of the U fibers and the initial modulus of the U/H blend fibers was slightly lower than that of the U fibers when the hot-draw ratio was less than 3. The tensile strength of the U/H blend fibers was higher than that of the U fibers with a hot-draw ratio less than 3 because the fibers are stretched at room temperature during the tensile testing process, and the microstructures change when the fibers break. The orientation of the molecular chains, the formation of crystalline regions, and the elongation of the grains’ shape occur mainly in the process of the draw ratio increasing from 3 to 9. At this stage, more extended molecular chains are formed, a more amorphous phase is transformed into a crystalline phase, more crystal structures are oriented in the axial direction, and grains are stretched to be slender and are compacted closely. The more compact structure can enhance the van der Waals force between macromolecular chains, and the slippage between molecular chains is prevented. Thus, the initial modulus and tensile strength increased rapidly and the elongation at break decreased rapidly as the hot-draw ratios increased from 3 to 9. As the hot-draw ratios increased from 9 to 15, the extension of the molecular chains and the evolution of the crystal structures slowed down. Therefore, the initial modulus and tensile strength increased slowly and the elongation at break decreased slowly as the hot-draw ratios increased from 9 to 15. The improvement effect of the HDPE on the microstructure of the blend fibers was reflected as the hot-draw ratios increased from 3 to 15.

## 4. Conclusions

In the spinning process, molecular chains slipped as the initial drawing process proceeded. The relatively short molecular chains of HDPE can lubricate the extremely long molecular chains of UHMWPE. The molecular chain orientation degree of the U/H blend as-spun filaments and the U as-spun filaments increased from 6.89 to 41.65 and from 4.68 to 30.56, respectively. The initial oriented molecular chains in the amorphous phase formed crystals. The crystallinity of the U/H blend as-spun filaments and the U as-spun filaments increased from 56.28% to 58.01% and from 52.47% to 54.58%, respectively. The crystal grains were shortened in the radial direction and elongated in the axial direction because of the slippage of the molecular chains in the drawing direction. *L*_110_, *L*_200_, and *L*_020_ of both filaments decreased, and *L*_002_ of both filaments increased as the initial draw ratios increased from 1 to 9. 

In the hot drawing process, chain segments were oriented in the drawing direction. The molecular chain orientation degree of the U/H blend fibers and the U fibers increased rapidly from 41.65 to 95.47 and from 30.56 to 94.58, respectively, as the hot-draw ratios increased from 1 to 9. The crystallinity of both the U/H blend fibers and the U fibers increased significantly because of the chain segment orientation. The crystallinity of the U/H blend fibers and the U fibers increased from 58.01% to 64.02% and from 54.58% to 62.53%, respectively, as the hot-draw ratios increased from 1 to 9. The grains of the fibers became slender. The *L*_200_, *L*_020_, and *L*_002_ of the U/H blend fibers were 10.2, 10.5, and 37.8 nm, respectively. The *L*_200_, *L*_020_, and *L*_002_ of the U fibers were 12.5, 10.3, and 35.7 nm, respectively. Crystal structures were oriented in the drawing direction. The *f*_c_ values of the U/H blend fibers and the U fibers increased from 0.72 to 0.95 and from 0.65 to 0.93, respectively, as the hot-draw ratios increased from 1 to 6. The tensile strength and initial modulus were improved and the elongation at break of the fibers decreased because of the slender and compact crystal structure. The tensile strength of the U/H blend fibers and the U fibers increased from 389 to 1130 MPa and from 304 to 832 MPa, respectively, and the initial modulus of the U/H blend fibers and the U fibers increased from 1.1 to 20.5 Gpa and from 1.5 to 17.7 GPa, respectively, and the elongation at break of the U/H blend fibers and the U fibers decreased from 318% to 12% and from 287% to 28%, respectively, as the hot-draw ratios increased from 1 to 15. Blending with HDPE could result in a higher orientation degree, an increase in the crystallinity, and a more elongated crystal grain structure of the blend fibers. Therefore, the mechanical properties of the U/H blend fibers were better.

## Figures and Tables

**Table 1:** Parameters of UHMWPE/HDPE blend.

Groups	Mass Ratio of UHMWPE/HDPE	MFI of HDPE (g/10 min)
U/H	6:4	0.9
U	10:0	N/A

**Table 2:** Take-up speeds of the winder and as-spun filament diameters with different initial drawing ratios.

Initial drawing ratio	Take-up speed (m/min)	As-spun filament	As-spun filament diameter (μm)
1	0 (Free fall)	U/H	588 ± 10
		U	597 ± 11
3	2.5	U/H	346 ± 8
		U	353 ± 10
6	5	U/H	242 ± 7
		U	245 ± 8
9	7.5	U/H	199 ± 6
		U	202 ± 5

**Table 3:** Fiber diameters with different hot-draw ratios.

Hot-draw ratio	Fiber	Fiber diameter (μm)
1	U/H	199 ± 6
	U	202 ± 5
3	U/H	115 ± 5
	U	116 ± 5
6	U/H	82 ± 3
	U	83 ± 4
9	U/H	66 ± 5
	U	67 ± 4
12	U/H	58 ± 3
	U	58 ± 4
15	U/H	51 ± 2
	U	52 ± 3
